# Downregulation of a Phi class glutathione *S*-transferase gene in transgenic torenia yielded pale flower color

**DOI:** 10.5511/plantbiotechnology.24.0409a

**Published:** 2024-06-25

**Authors:** Misako Akagi, Noriko Nakamura, Yoshikazu Tanaka

**Affiliations:** 1 Research Institute, Suntory Global Innovation Center Ltd.

**Keywords:** anthocyanin, flower color, glutathione *S*-transferase, RNAi, vacuole transport

## Abstract

The members of glutathione *S*-transferase (GST) belonging to the Phi class of the GST family are known to play a role in anthocyanin transport to the vacuole. We isolated a GST orthologue from the torenia petal cDNA library. Transgenic plants transcribing GST double stranded RNA were generated from a torenia cultivar having blue flowers. These plants exhibited a range of flower colors, from blue to almost white. Quantitative RT-PCR confirmed the downregulation of the GST transcript, accompanied by a decrease in anthocyanin levels in the petals of the transgenic plants, whereas flavone levels remained unchanged. These results suggest that GST is involved in anthocyanin transport in torenia petals, and that anthocyanins and flavones are likely transported to the vacuole through different mechanisms.

Flavonoids and their colored class of compounds, anthocyanins, confer a wide spectrum of flower colors, from pale yellow to red and blue. Flower color is a highly valued trait in floricultural crops. Anthocyanins also determine the color of fruits and seeds. The flavonoid biosynthetic pathway is evolutionarily conserved among seed plant species and one of the most well-characterized in plants (Tanaka et al. 2008). Flavonoids are typically synthesized in the cytosol and transported to vacuoles via three mechanisms; (1) glutathione *S*-transferase (GST)-mediated ATP dependent ABCC type transporter, (2) proton gradient dependent multidrug and toxic compound extrusion (MATE) *trans*-porter, and (3) vesicle-mediated mass transport (Kaur et al. 2021; Pucker and Selmar 2022). These three mechanisms are not mutually exclusive and can co-exist in the same cell.

The involvement of GST in anthocyanin transport was first discovered in maize (*Bronze 2* (*Bz2*)) (Marrs et al. 1995), followed by petunia (*Anthocyanin 9* (*An9*)) (Mueller et al. 2000), *Arabidopsis* (*trans-parent testa* 19 (*TT19*)) (Kitamura et al. 2004) and many other plants. The genomes of plant species encode 50–100 GSTs, which are classified into 14 classes (Cao et al. 2022). The majority of anthocyanin-related GSTs (arGSTs) belong to the Phi class, with the remainder belonging to the Tau class (Tasaki et al. 2020). The mechanism of arGST in anthocyanin transport has recently been elucidated. For example, the cotton GST GhTT19 has been shown to interact with cyanidin 3-glucoside (Chai et al. 2023). The ABC transporters, VvABCC1 and AtABCC2, have been shown to co-transport cyanidin 3-glucoside and glutathione (Behrens et al. 2019; Francisco et al. 2013). Behrens et al. (2019) demonstrated that AtABCC2 transports anthocyanidin 3-glucosides, luteolin/apigenin 7-glucosides, and kaempferol/quercetin 3-glucosides. Conversely, AtABCC1, AtABCC2 and AtABCC14 can uptake acylated anthocyanins independently of glutathione (Dean et al. 2022). Our understanding of anthocyanin transport to vacuoles and the role of GST in flavonoid transport is still limited. Recent studies have shown that poplar GST8 catalyzes the formation of anthocyanidins from flavan-3, 3, 4-triol, a product of anthocyanidin synthase, suggesting that GSTs also function as enzymes in anthocyanin biosynthesis (Eichenberger et al. 2023).

Functional analysis of arGSTs has been achieved by mutant complementation (Alfenito et al. 1998; Kitamura et al. 2004, 2012; Sasaki et al. 2012) or downregulation of *GST* genes in transgenic plants. In a previous study, CRISPR/Cas9 knockout of the Reduced Anthocyanins in Petioles (*RAP*) gene encoding a GST in strawberry resulted in white-fruit and green stem phenotypes, whereas *RAP* overexpression increased anthocyanin levels (Gao et al. 2020). CRISPR/Cas9-mediated gene editing of *GST1* in *Gentiana triflora* resulted in gentian plants with nearly white flowers containing <10% of the anthocyanin levels in the host plant (Tasaki et al. 2020). CRISPR/Cas9 knockout of *OsGSTU34*, a gene encoding *Bz2*-like Tau family GST in black rice, has been found to result in a substantial decrease in cyanidin 3-glucoside and peonidin 3-glucoside levels (Mackon et al. 2023). CRISPR/Cas9 knockout of *DcGST1* has been reported to result in orange taproots instead of purple ones (Duan et al. 2024).

Herein, we selected torenia as a model plant (Aida 2009) to examine the roles of GST in flavonoid biosynthesis and flower color. The petals of torenia accumulate flavones such as apigenin and luteolin lucosides, and various anthocyanins, mainly malvidin 3-glucoside-5-coumaroyl glucoside (Nakamura et al. 2010). We have previously achieved color modification of torenia plants by silencing the expression of anthocyanidin synthase, flavonoid 3′, 5′-hydroxylase, and other genes using double-stranded-mediated interference (RNAi) (Nakamura et al. 2006, 2010). In this study, we extended the application of RNAi technology to downregulate arGST in vivo to identify its function and evaluate its potential as a target for flower color modification.

A screening of the torenia petal directional cDNA library (Ueyama et al. 2002) using the *Ipomoea nil* GST gene (INIL02g39744) yielded many hits. A dozen of these hits were recovered, and nine sequences from their 5′-ends were obtained, with eight of them being homologous to the *I. nil* GST. Their nucleotide sequences were almost identical but were classified into two groups (one group including TGST#10 and the other). They showed >97% identity in the sequenced region (approximately 400 bp). These sequences are the only *GST* isolated by the library screening using the *I. nil* arGST as the prove although a plant species known to is contain 50–100 kinds of GST (Cao et al. 2022). One of them, TGST#10, was completely sequenced (DDBJ/EMBL/GenBank accession number LC78559). TGST#10 encoded a full-length amino acid sequence of 215 residues with a molecular weight of 24,339.45 Da. The amino acid sequence of TGST#10 exhibited 60%–70% identity to reported dicot arGSTs. A phylogenetic tree of GSTs was constructed based on amino acid sequences using CLUSTALW (https://www.genome.jp/tools-bin/clustalw (Accessed Feb 27, 2024)) and Dendroscope software (Huson and Scornavacca 2012). TGST#10 belongs to the Phi class of GSTs (Figure 1), whereas monocot GSTs such as Bz2 were found to belong to the Tau class. It is reasonable to infer that torenia TGST#10 is closely related to perilla, sesame, and *Buddleja alternifolia* GSTs, as all four species belong to the order Lamiales.

**Figure 1:**
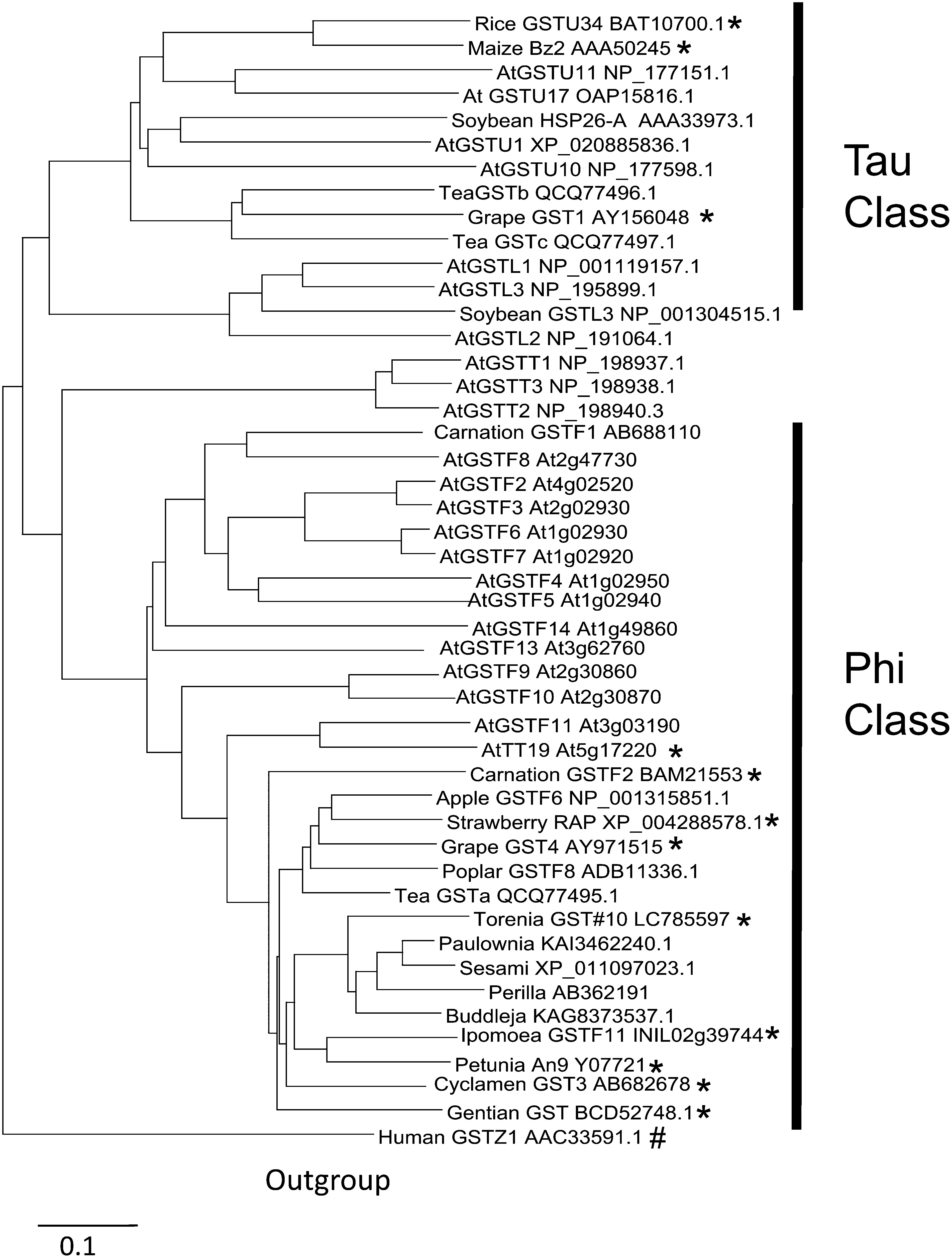
Figure 1. Phylogenetic tree of anthocyanin-related glutathione *S*-transferases (arGSTs). Plant names and database accession numbers are shown (At, *Arabidopsis thaliana*). The TGST#10 sequence from torenia (analyzed in this study) belongs to the Phi class of the GST family. Sequences experimentally verified to be involved in coloration by anthocyanin are shown with *. Human GSTZ1 (#) was used as the out group as reported (Tasaki et al. 2020).

A binary vector transcribing TGST#10 double-stranded RNA (RNAi) based on a bock bone vector, pBin PLUS (van Engelen et al. 1995), was constructed via restriction enzyme digestions (Figure 2A). El_2_35S, an enhanced cauliflower mosaic virus 35S promoter (Mitsuhara et al. 1996), was used to transcribe *TGST#10*. The binary vector was introduced into the plants of torenia cultivar Summerwave blue (Suntoryflowers Ltd., SWB), an interspecies hybrid of *Torenia fournieri* and *T. concolor*, as previously described (Aida 2012) using *Agrobacterium tumefaciens* Agl0 (Lazo et al. 1991). More than 50 transgenic plants were generated. No completely white flowers were obtained. Most of the transgenic plants had a paler flower color than the host, with a range of color intensities. The transgenic plants were classified into four groups based on flower color intensity. Two lines from each group (Figure 2B, T-2 and T-10, T-6 and T-9, T-20 and T-27, and T-19 and T-21) were selected for quantitative real-time PCR (qPCR) using an ABI7000 sequence detection system (Applied Biosystems Inc.) following a previously described method (Nakamura et al. 2006, 2010). The total RNA from the petals of buds was prepared for qPCR of TGST#10 transcripts using a set of primers (forward primer (FP), 5′-ATTTGGTCAAGTTCCTGCAATAGA-3′; reverse primer (RP), 5′-TTATTGCCCTGGATTCGAAAA-3′; TaqMan probe, 5′-ACGGCGATTTCAAG-3′). To standardize reactions, torenia glyceraldehyde-3-phosphate dehydrogenase gene was used as a control (FP, 5′-GCATTGAGCAAGACGTTTGTG-3′; RP, 5′-ACGGGAACTGTAACCCCATTC-3′; TaqMan probe; 5′-AGCTTGTGTCGTGGTACG-3′) as previously reported (Nakamura et al. 2006). The reactions were performed in triplicate however as for TT94-6 and TT94-27, only two measurements were successful. As shown in Figure 2C the amount of TGST#10 transcripts was significantly reduced in the transgenic plants compared to the control plants especially in the lines with paler flower color.

**Figure 2:**
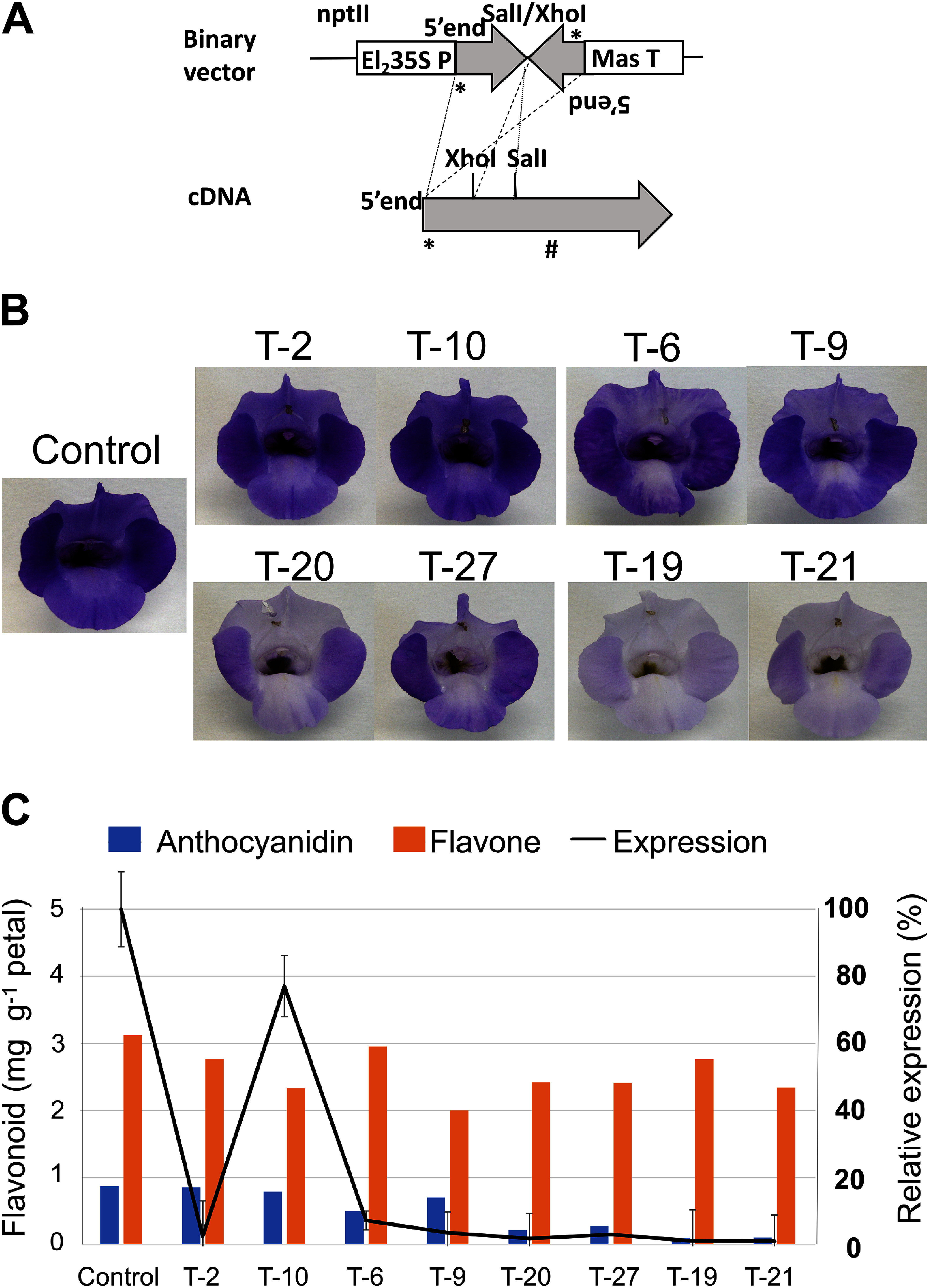
Figure 2. Downregulation of TGST#10 gene in transgenic torenia. A. Schematic representation of the construction of the binary vector used to downregulate TGST#10. The internal *Sal*I and *Xho*I restriction sites of the cDNA (*, initiation codon; #, stop codon) were used to generate the fragments for the construction. The inverted repeat and loop sequences deriving from the 5′ portion of the cDNA are approximately 300 bp and 180 bp in length, respectively. The enhanced cauliflower mosaic virus 35S promoter (El_2_35S P) and the manopaline synthase (Mas T) terminator were used to regulate the expression of the inverted repeat and loop sequences. Neomycin phosphor transferase II (nptII) was the selectable marker. The length is not drawn to scale. B. The flowers of the selected lines of the four groups classified on the bases of flower color intensity. C. Total quantities of anthocyanidins and flavones of the selected transgenic lines. The compositions of anthocyanidins and flavones are shown in Supplementary Table S1. The relative TGST#10 transcript levels is also shown (the black line).

Anthocyanidin and flavone analysis was carried out described before (Nakamura et al. 2010). The petals of SWB plants contained mainly malvidin, followed by peonidin, as anthocyanidins. Malvidin and peonidin were also the most highly accumulated anthocyanidins in transgenic plants, indicating that the downregulation of TGST#10 did not affect the anthocyanidin species accumulated in the petals (Supplementary Table S1). Petals with paler colors had lower levels of anthocyanidin. T-19 and T-21 contained 12% and 11% of the host anthocyanin, respectively (Figure 2C, Supplementary Table S1). These results confirm the key role of arGST in anthocyanin accumulation and flower color as previously reported (Sasaki et al. 2012; Tasaki et al. 2020). It is curious that T-2 petals contain a comparable amount of anthocyanidins to the host and T-2 flower color is only slightly paper than the host despite downregulation of TGST#10 transcripts. Further studies are necessary to clarify the reason. Absence of pure white transgenic flowers is comparable to a previous report wherein CRISPR/Cas9 was used to edit *GST1* in gentian plants to yield pale but not white flowers (Tasaki et al. 2020). Carnation arGST (*DcGSTF2*) mutant has a pale flower color (Sasaki et al. 2012) whereas petunia *an9* mutant has a white flower (Alfenito et al. 1998). Our results regarding torenia in the present study might suggest the presence of alternative or spontaneous pathways to transport anthocyanins into vacuoles. GST other than TGST#10 such as Tau class GST might partly complement the downregulation of *TGST#10*. Knock out of *TGST#10* in torenia by genome editing may clarify further its roles in anthocyanin transport or biosynthesis. Some plant species have redundant mechanisms for anthocyanin transport or anthocyanidin biosynthesis (Kaur et al. 2021; Pucker and Selmar 2022; Tanaka et al. 2008). In case arGSTs is the enzyme catalyzing anthocyanidin biosynthesis, as recently suggested (Eichenberger et al. 2023), spontaneous conversion to anthocyanidin may be possible.

In contrast, the levels of flavones did not change significantly in the paler colored petals. T-19 and T-21 also had approximately 80% of the flavones of the host (Figure 2C, Supplementary Table S1). These results suggest that TGST#10 plays a critical role in anthocyanin accumulation but not in flavone accumulation. This study could not determine whether arGST or TGST#10 is involved in anthocyanin transport, anthocyanidin biosynthesis, or both. Extensive metabolome analysis of transgenic plants may contribute to elucidating the true function of arGST.

Specificity of the transport has been studied to some extent. TT19 in *Arabidopsis* is involved in the transport of anthocyanins and proanthocyanidins to the vacuole, whereas *Medicago* TT19-like GSTs (MtrGSTF7 (Panara et al. 2022) and MtGSTF7 (Wang et al. 2022)) and RAP in strawberry (Gao et al. 2020) are not involved in proanthocyanidin transport. In *Vitis* VvGST4 transports anthocyanins and proanthocyanidins to the vacuoles, whereas VvGST1 and VvGST3 transport only proanthocyanidins (Pérez-Díaz et al. 2016). Understanding how arGST or TGST#10 recognizes anthocyanins with diverse structures poses an intriguing question. This, along with elucidating the genuine function of arGST in vivo, presents a promising challenge for future research.

## Abbreviations

arGSTs: anthocyanin-related GSTs
GST: glutathione *S*-transferase
qPCR: quantitative real-time PCR
