# Supplementary material for: Downregulation of a Phi class glutathione S-transferase gene in transgenic torenia yielded pale flower color
Source: Plant Biotechnol (Tokyo). 2024 Jun 25;41(2):147–51. doi: 10.5511/plantbiotechnology.24.0409a (PMC11500598; doi:10.5511/plantbiotechnology.24.0409a)
Supplement: Supplementary Data [file plantbiotechnology-41-2-24.0409a-s001.pdf]

Supplementary Table S1. Flavonoid compositions of the torenia host (control) and transgenic lines (μg g-1 wet petal)

	Anthocyanidin								Flavone			
	Total Anthocyanidin	Relavive Content (%)	Malvidin	Peonidin	Pelargonidin	Petunidin	Cyanidin	Delphinidin	Total Flavone	Relavive Content (%)	Luteolin	Apigenin
Control	867	100.0	491	299	2.1	40.0	34.0	0.5	3125	100.0	675	2450
T-2	853	98.4	523	271	1.1	35.6	22.3	0.4	2772	88.7	612	2160
T-10	783	90.3	414	312	1.4	28.6	26.8	0.3	2335	74.7	615	1720
T-6	494	57.0	318	137	0.5	26.8	11.3	0.4	2953	94.5	733	2220
T-9	698	80.5	423	229	1.1	29.2	15.1	0.4	2006	64.2	496	1510
T-20	215	24.8	145	48.1	0	15.9	5.1	0.5	2418	77.4	558	1860
T-27	270	31.1	190	55.1	0	19.1	5.3	0.5	2414	77.2	634	1780
T-19	92.3	10.6	66.7	13.9	0	9.3	2.1	0.3	2767	88.5	707	2060
T-21	98.8	11.4	69.3	15.5	0	10.6	2.9	0.5	2341	74.9	581	1760
